# Necessary but not sufficient? Engaging young people in the development of an avatar‐based online intervention designed to provide psychosocial support to young people affected by their own or a family member's cancer diagnosis

**DOI:** 10.1111/hex.12473

**Published:** 2016-06-12

**Authors:** Ceri Phelps, Masoumeh Minou, Andrew Baker, Carol Hughes, Helen French, Wayne Hawkins, Andrew Leeuwenberg, Rebecca Crabtree, Paul B. Hutchings

**Affiliations:** ^1^ School of PsychologyUniversity of Wales Trinity Saint DavidSwanseaUK; ^2^ Tenovus Cancer CareCardiffUK

**Keywords:** avatar counselling, cancer, intervention development, online counselling, young people

## Abstract

**Objective:**

This study discusses the challenges and successes of engaging young people in a project aimed at developing an online counselling intervention for young people affected by cancer.

**Context:**

For younger people with a diagnosis of cancer or who are caring for someone with cancer, the psychosocial consequences can create significant challenges for their social and educational development. Whilst young people have been shown to be reluctant to make use of traditional face‐to‐face counselling, research is beginning to suggest that effective therapeutic relationships can be formed with young people online.

**Design:**

The first phase of the study involved working with a ‘Young Persons’ Panel’ of healthy school pupils and university students to develop and pilot an online counselling intervention and study materials in preparation for a pilot evaluation of the intervention.

**Intervention:**

An avatar‐based virtual reality counselling world was created where young people can create their own avatar and receive counselling over the Internet from a qualified counsellor via an avatar in a virtual reality world.

**Findings:**

The process of engaging young people in the C:EVOLVE project enabled a unique intervention to be developed and demonstrated positive developmental opportunities. However, despite the rigorous approach to the development of the intervention, initial attempts within the pilot evaluation phase of the study showed difficulties recruiting to the study, and this phase of the study has currently ceased whilst further exploratory work takes place.

**Conclusion:**

This study has demonstrated the complexities of intervention development and evaluation research targeted at young people and the challenges created when attempting to bring clinical practice and research evaluation together.

## Introduction

The negative psychosocial impact of a cancer diagnosis is well documented in the psycho‐oncology literature. These psychological sequelae extend beyond the affected individual to the family and the wider support network and have potentially long‐term consequences on quality of life, social and occupational functioning.^1^, ^2^, ^3^ For younger people with a diagnosis of cancer, the wider psychosocial and educational consequences can be even more complex, creating significant communication and interpersonal difficulties within existing support networks^4^, ^5^, ^6^ that can have a longer‐term impact on their social and educational development that can last into adulthood.^7^, ^8^, ^9^, ^10^, ^11^ Young people can also be indirectly impacted by a cancer diagnosis through coping with the diagnosis of another family member and may find it hard to cope with the emotional burden of experiencing a family member struggle with their diagnosis and the informal caring role that may ensue. Studies have shown that the impact of parental cancer on children can create psychosocial difficulties in vulnerable young people.^12^, ^13^, ^14^, ^15^ Clearly, therefore, identifying appropriate support mechanisms for younger people either personally affected or with affected family members is an important element of the care pathway for such individuals and their subsequent psychosocial well‐being.^16^

Whilst a growing number of studies are evaluating interventions designed to help young people affected by cancer,^17^, ^18^, ^19^ there has been little documented success in relation to the provision of counselling services for such young people who are often reluctant to engage in more traditional forms of counselling due to concerns about stigma.^20^, ^21^, ^22^ In the counselling field, the provision of support over the Internet has become an increasingly popular alternative to the more traditional face‐to‐face delivery of counselling, offering the user a perception of a sense of privacy and anonymity that cannot be accessed by traditional counselling without the constraints of travel, time and location.^23^, ^24^, ^25^ It is encouraging therefore that research is beginning to suggest that effective therapeutic relationships can be formed with young people online and that it is a service which can be valued and used by young people.^4^, ^26^, ^27^, ^28^ The use of online interventions as a mechanism through which to achieve psychological benefit amongst young people across a wide range of settings is reporting promising outcomes.^29^, ^30^ Specifically, engagement with avatars is recognized as being familiar to most young people who are used to engaging with virtual reality computer games, and younger age groups affected by cancer have been found to report positive experiences from engaging with online counselling.^27^, ^28^ Whilst there remain concerns about the ability to form effective therapeutic relationships online in contrast to more traditional face‐to‐face counselling,^29^ evidence is showing that effective relationships can be established online, with a growing number of professionals willing to embrace such advances if given appropriate training and support.^30^, ^31^, ^32^

Clearly, the provision of any online counselling or psychosocial support intervention to a vulnerable group must overcome a number of ethical and logistical challenges. Significant considerations in offering any form of online psychological support to vulnerable groups include those of confidentiality and data protection, respect for the autonomy and dignity of the participants, valid consent processes, and access to follow‐up care.^33^, ^34^, ^35^ In the context of the provision of online services to young people potentially under the age of 18, these challenges also include the safeguarding of young people in an online environment, recognizing the boundaries of appropriate provision to minimize potential harm, gaining parental consent and putting steps in place to counteract any technical interruptions.^33^, ^34^, ^35^, ^36^ In designing evaluation studies that assess the potential benefits of such interventions, therefore, there are a number of significant issues that need to be built into the methodology and recruitment process of the research to ensure an ethically robust study that protects the well‐being of its young participants at all times. Given the logistical and ethical challenges in the provision of such practice, therefore, developing not only an intervention but a recruitment protocol that is attractive to both young people and parents is a key challenge.^37^, ^38^ A growing body of literature already recognizes the challenges identified above and the importance of the role of not only young people as research partners to better understand youth and their specific environments but also of involving key stakeholders in the development of such interventions. Specifically, researchers, policymakers and programme evaluators have begun to engage young people as research partners, not only to better understand youth and the environments that impact their development, but also to provide them with the tools to develop and validate knowledge and to influence the development of programmes and policies designed to affect their lives.^39^, ^40^, ^41^, ^42^, ^43^

This study reports on a study (the C:EVOLVE project) designed to develop an avatar‐based online counselling world for young people affected by cancer in some way in their lives, and discusses the challenges and benefits of engaging young people themselves in the development of the intervention. Fundamental to the development of the intervention was the engagement of young people throughout the process, both in the initial development of the intervention and in its subsequent evaluation.

## Project aims and methodology of the C:EVOLVE project

The overall aims of this project were threefold: to develop a virtual reality avatar‐based counselling world for young people affected by cancer informed by the young people likely to make use of such a service, to gain pilot data on the acceptability, likely usage and therapeutic value of the intervention, and to identify recommendations regarding the potential pitfalls and challenges in relation to the future delivery of such online interventions for young people. Phase I of the project represented the initial planning, development and piloting of the online counselling world, with Phase 2 representing the pilot evaluation phase. Both phases of the study received ethical approval from the University's School of Psychology and Counselling Research Ethics Committee and the South West Wales NHS Research Ethics Committee. Alongside this, an appropriate project team was recruited which included young computer design and programming research assistants who were current students at the university, overseen by an appropriately experienced project steering group including the research team and qualified counsellors.

### Recruitment to the Young Persons’ Panel

Advertisements for members of the Young Persons’ Panel were posted on the university's website and virtual learning platform and sent to three local schools. Four student volunteers aged 19–23 years (two males and two females of White British nationality) were recruited from the School of Psychology and Counselling at University of Wales Trinity Saint David, Swansea, who were studying for a degree in Counselling Studies and Psychology alongside a Certificate in Counselling Skills. These students were recruited carefully following an interview assessing maturity, ethical sensitivity and a willingness to provide a mentoring role to the younger individuals in the project group. An additional eight volunteer pupils from a local school were then identified by the school's deputy headmaster and the relevant consent forms (including parental consent for those under 18 years of age) were obtained. These school pupils ranged in age from 15 to 18 years, with four males and four females, and five of White British ethnicity, one of Asian ethnicity, one of Black African ethnicity and one of mixed Welsh–Turkish ethnic origin. A key outcome of these early deliberations was the decision not to specifically target young volunteers to the Young Persons’ Panel with a diagnosis of cancer or caring for someone with cancer for the development phase of the project given the ethical sensitivities in the early stages of this project. All of the young people were expected to demonstrate an awareness of, and sensitivity to, the issues that may be faced by young people affected by cancer in some way in their lives and to also have some experience in using virtual worlds. The Young Persons’ Panel (YPP) final composition therefore consisted of 12 young people between the ages of 15 and 23. One student was identified as the YPP lead and was also asked to attend the project steering groups to represent the YPP.

### Engaging young people in the C:EVOLVE Young Persons’ Panel

The YPP met on a monthly basis on university premises during the first 6 months of the project. The first two project meetings included educational sessions about the ethics of counselling young people and the sensitivities of the project target group alongside the ground rules and boundaries of the YPP. During the meetings themselves, activities were structured to include informal testing and feedback of the intervention as it developed alongside more structured feedback questionnaires on the usability and likeability of the interface and study materials and focused smaller‐group activities moderated by the older members of the YPP and overseen by the lead researcher and project research assistant. Activities were structured by the project team to ensure that the YPP were able to provide contributions and feedback to all elements of the project, including the cosmetic and functional elements of the intervention and the study materials being prepared for the pilot evaluation study.

In addition to the monthly meetings, a range of approaches were employed to engage the young people throughout the project, one of the most effective ways of maintaining regular contact with the YPP being the use of a closed Facebook page. This Facebook page was moderated on a daily basis by the lead researcher and project Research Assistant, but the YPP team leader was also given the rights to moderate and edit any Facebook posts. In the early stages of the project, the Facebook page was used to primarily remind the YPP of the next meeting, but once the group appeared more comfortable with each other it was then used as an effective communication channel for obtaining feedback on important issues between meetings.

### The development of the intervention

The YPP were involved in a number of activities that included brainstorming activities at the start of the project to identify important elements of a virtual reality counselling world; voting on preferences for avatar design and related assets, for example preferred facial features and types of clothing; identifying the range of relevant emotions, emoticons and animations that YPP would expect to be able to select in an online counselling world; and assisting in the creation of the Phase II ‘Frequently Asked Questions’ page for technical help/guidance.

The YPP were involved in the on‐going testing of the C:EVOLVE world as it developed, providing oral and written feedback on the usability and acceptability of the intervention and engaging in practical testing of the software as it was being developed. This initial testing and feedback included creating and testing the avatars, attempting to break the code used by the programme developers, and using the emoticons and emotion displays to convey changing emotional responses. As the intervention developed, the YPP engaged in pseudo‐counselling sessions engaging in synchronous real‐time chat (with one young person in the role of counsellor and the other in the role of client) and replicated the process that participants would be working through for the Phase II evaluation (including testing out the links to the online survey). Towards the second half of Phase I, the YPP also provided input into materials being prepared for the evaluation in Phase II, including identifying appropriate questions for the ‘Frequently Asked Questions’ page and providing feedback on the readability of the recruitment materials and questionnaires.

Examples of the effective use of the closed Facebook page as a communication tool included asking the YPP to vote on their preferences for the preferred type of avatar face via Facebook and to provide comments on the readability of the ‘Frequently Asked Questions’ sheet in preparation for Phase II of the project. The closed Facebook page also served as an effective tool for organizing monthly meetings, gaining feedback, and providing updates on the progress of the virtual reality world.

### Final design of the ‘C:EVOLVE’ world

**Figure 1:**
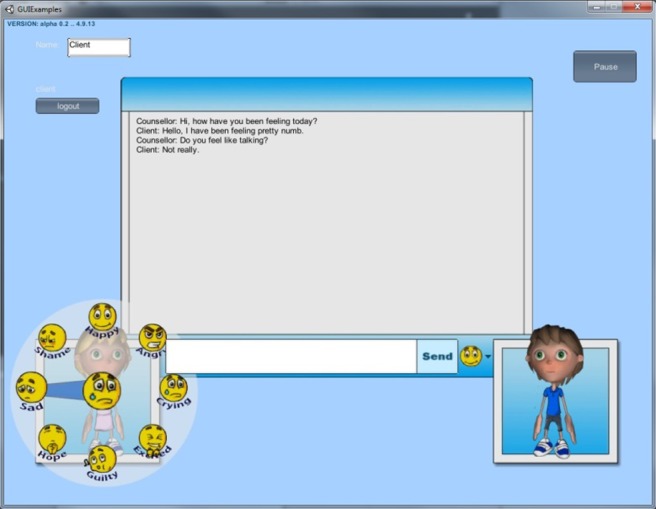
Screenshot of intervention interface.

The final C:EVOLVE world allows its users (both client and counsellor) to create an avatar; a 3D graphical representation of the individual can be customized through altering and/or adding elements such as hair, clothing and skin tone. The online session itself is based around the use of synchronous real‐time chat supported by full‐body emotion displays through the avatar and the use of emoticons through which participants are able to select stable and fluctuating emotions at any point during the counselling session to express a range of core feelings, for example sad, happy, as well as ‘flash’ feelings such as confused, scared and worried. Figure 1 demonstrates a screenshot of the final C:EVOLVE world.

## Evaluation of the success of engaging young people in the C:EVOLVE project

### Reflections from the research team

Unsurprisingly, it took time for the YPP to form a cohesive group and actively engage in the project from the outset, with the younger members of the YPP in the early meetings sometimes appearing reluctant to contribute to the discussions. However, when the younger group members worked in collaboration with the university students, their responses became much more specific and detailed. An interesting observation quickly formed by the project team was that these younger individuals became more enthused and animated when able to test out the developing interface and were in fact highly vocal and critical when asked to provide feedback on the intervention itself. Whilst these younger group members tended to focus on the technical elements of the intervention (the ‘breakability’ of the system, the quality of asset design, etc.), the older members of the YPP appeared to have a more balanced view of the wider intervention (e.g. how easy it was to use and navigate). The older members of the YPP also played an important mentoring role within the various activities, often being asked to take the lead to encourage discussions and input from all involved in specific activities.

### Thematic analysis of YPP's evaluation data

At the end of their involvement with Phase I of the project, the YPP were asked to provide feedback on their experience of being part of the C:EVOLVE project, through an evaluation questionnaire and the provision of free‐text comments in the form of written comments. Six questions centred around the perceived benefits and challenges of being part of the project (e.g. What is the most important thing you liked about your participation with the C:EVOLVE project?). A free‐text questionnaire was provided to participants asking them to respond to the questions in their own words. The data were subjected to a basic thematic analysis^44^ lead by the project research assistants and validated with the project lead. This analysis suggested that the young people appeared to find the experience very positive and rewarding in a number of ways, with three overarching themes identified: the development of interpersonal and team‐working skills; making a difference; and empathy and understanding. Few negative comments about the involvement in the project were reported, with the more negative comments relating to procedural elements of their involvement; one participant for example stated that they would have liked to have met more often, whilst three of the young people mentioned that they did not like their photographs taken.

#### The development of interpersonal and team‐working skills

In line with the research team's own observations as the project progressed, participants reported a number of benefits from their involvement in the project in relation to the development of their interpersonal, communication and team‐working skills:

It is something that I haven't done before and it helped me develop new skills [M1, 16]

For a number of the young participants, these benefits included confidence and communication skills developed as a consequence of learning how to work as part of a team:

The benefits for me are: being able to work as part of a team and to speak to young people [M5, 23]

Being part of this project gave me more confidence and enabled me to express my opinions in a team [M2, 18]

What I liked about the project is working with others, that has improved my communication skills. [F2, 18]

Interestingly, a number of participants also commented on the wider benefits of exposure to a university‐based research environment in the development of their wider experiences and awareness:

Being involved with research projects and universities broadens the way you look at the world, you become more aware. [F4, 18]

You get a better idea of university life, meet new people, work in a different way and see things in real life. [M4, 23]

Getting involved in research opportunities makes you an all round person [M2, 18]

#### Making a difference

An overriding theme that emerged from much of the analysis of the evaluation data suggested that one of the reasons why the young people chose to volunteer for this project was the opportunity to make an active contribution to a project that they clearly believed could make a difference to young people affected by cancer in some way in their lives.

I liked the development of the programme and seeing that the ideas I have put forward are being implemented in the project. [M2, 18]

For one of the younger male participants, the experience of being involved in a project of this nature clearly evoked a positive emotional response in him:

It feels very good, I haven't done anything like this before and helping people makes me feel great. [M1, 16]

Female participants appeared to specifically value the opportunity to become involved in a project in its infancy which may have a positive impact on other young people's lives.

I liked the chance I was given to express my opinion and make a difference. [F4, 18]

I liked meeting new people and being able to help in a project to benefit others. [F1, 22]

We saw something built from the start, and if it's a success it will be nice to know we helped to create it [F5, 22]

I feel great to be part of such a great cause which gives young people affected with cancer a great opportunity to get help. [F1, 22]

I feel happy that I have been given the opportunity. I'm relieved that my input can improve someone's wellbeing. [F6, 18]

Finally, responses to the question asking why young people should be involved in research projects demonstrated a good understanding of the benefits young people can bring to the development of interventions of this nature:

Younger generations will understand how to appeal to their age group and with technology around the project more effectively and successfully than those of other ages [F2, 18]

Because it will be more effective if aimed at young people and designed by them. [F3, 22]

#### Empathy and understanding

Two of the female participants highlighted that they had developed an understanding of the skills needed when offering people counselling support, whether face‐to‐face or online:

I've learnt the importance of dealing with issues surrounding cancer sensitively [F4, 18]

I've learnt that we should try to help a client without overstepping the mark [F6, 18]

Another female participant reported a better understanding of not only the many ethical issues surrounded the development of such an intervention, but of both counselling and the use of social media more broadly:

It's helped me understand the ethics of counselling and using social media [F6, 18]

Other comments suggested a more general appreciation of what younger individuals affected by cancer in some way have to deal with in their day‐to‐day lives.

This project has given me an understanding of the vulnerability that some young people have in their lives [M3, 17]

I have benefited from this project because I had to think about other people and issues they may face, and how not everybody is as lucky as we are [F4, 18]

#### Overall experience in a ‘Tweet’

As shown in Table 1, participants were also asked to ‘sum up’ their experience of being involved in the project through each participant coming up with a ‘tweet’, followed by a group vote for the favourite tweet.

**Table 1:** C:EVOLVE Phase I ‘tweets’

#LetTheKeyboardDoTheTalking
The keyboard is your inner voice screaming to be heard #Silent
Changing the face of counselling #CThisEvolve
Feels good to get involved in something that can change someone's life #Proud
Saving the avatar, one virtual world at a time #SitDownTypeOut
Counselling liberates your personal worries, keyboards puts your voice into text #KeyboardCounselling
Let's see how this project evolves #FunTimes
Can't type fast enough #OnlineCounselling

### Summary of Phase II evaluation

Following NHS ethics approval for Phase II, the project team were actively involved in promoting the next phase of the project and took part in a number of recruitment activities including posters at local hospitals and a mail out through the funding body to its supporters. The target sample for this phase of the study was between 6 and 8 volunteers aged between 12 and 25 years, either personally affected by cancer or with a relative with cancer. Each participant would be offered up to 6 weeks of online counselling with a qualified counsellor, with measures taken before and after each online session. Following a rigorous consent process including the screening out of highly distressed individuals, eligible participants were asked to complete a battery of mood, emotion and psychological functioning questionnaires through an embedded online survey link before and after each online session. Rigorous safeguarding procedures were put in place in accordance with British Association for Counselling and Psychotherapy (BACP) guidelines on online counselling and the Welsh Assembly School‐Based Counselling policies (2011) to ensure good practice in the delivery of online counselling and the protection of young people within this virtual environment. These included the following:

1. Ensuring that online counselling was appropriate for the presenting concern through robust screening procedures.
2. Maintaining confidentiality and privacy in line with the Data Protection Act (2004) whilst making it clear that concerns over the welfare of the child will take precedence.
3. Minimizing harm and distress through the recruitment of an appropriately qualified counsellor and clear withdrawal, exit and follow‐up processes.
4. An identified process for following up the online session through telephone contact in the event of interruption of counselling due to technological problems.

In this 4‐month period, four expressions of interest were received; one was ineligible to take part due to being below 13 years of age, another young person was interested but his mother was reluctant for him to take part in online counselling; another never followed up the initial query, and one participant consented and agreed to take part.

The one participant who consented to take part completed the baseline measures and first online counselling session, but was then contacted us to say that she felt unable to continue with the study due to the passing away of her affected family member. Due to the funding period coming to an end, we have currently ceased active recruitment to the study, but are exploring opportunities to continue this evaluation phase.

## Discussion

The aim of the C:EVOLVE project was to develop an effective online counselling intervention for young people affected by cancer that was seen as relevant and acceptable by that target user group. The young people provided critical input during the development of the intervention and the design of the avatars to ensure that the package was easy to use, attractive and functional for the target group; this resulted in the development of a novel online counselling intervention which the project team considered ready to be piloted with young people affected by cancer in some way in their lives in the Phase II feasibility study. Unfortunately, despite the rigorous approach to the development of the intervention documented in this paper, initial attempts within the pilot evaluation phase of the study showed a poor level of recruitment to the study, with the project funding coming to close before any participant successfully completed the intervention.

Kime, McKenna and Webster^43^ have highlighted the essential role that young people can play in the development process of an intervention, and the 100% participant retention rate over the 6 months of the study suggests the young people did successfully engage with the project, with the use of the Facebook page appearing to facilitate this on‐going engagement.^45^ Previous research has highlighted the personal benefits for young people getting involved in research such as a greater understanding of responsibility and building of new skills,^46^, ^47^ and this paper suggests that the young people gained valuable experience and knowledge from participation in the project. Our young people clearly learned about the process of ‘doing research’, and as the project developed it was apparent that the young people deepened their wider understanding of different aspects of counselling and the ethical issues associated with research and young people. Importantly, our study suggests that when engaging young people in the development of interventions, it is effective to also appoint older ‘mentors’ to support the young group members to facilitate effective dialogue.

There are a number of interlinked possible explanations for the early failure to recruit into this study; firstly, the intervention may have been inappropriate for the target audience. This is unlikely given the steps documented in this current paper; however, future in‐house testing will explore further into how young people navigate/respond to key elements of the intervention, and how they respond to the word ‘counselling’. There is a suggestion in the literature that online counselling may be more acceptable to young people if offered as an adjunct to face‐to‐face counselling,^48^ thereby providing a more simple referral mechanism but limiting the potential reach of such an intervention. It has to be acknowledged that the members of the Young Persons’ Panel did not themselves have cancer. Whilst the project team recognized from the outset that young people affected by cancer may well have different needs, the cautious approach operated for Phase I was considered appropriate on ethical grounds given the novelty of the project and the need to ensure that the intervention was acceptable to young people. On reflection, whilst the project steering group included counsellors with significant experience in counselling young people affected by cancer, engaging with young adults who had been through the cancer experience may have provided additional important insight into the development of the intervention and its evaluation.

Skinner & Latchford^49^ identified poor uptake rates for online forms of counselling that has also been documented elsewhere, and this reluctance and suspicion of e‐forms of therapeutic intervention may well also apply to a target population who we assumed would be more accepting of such interventions. Previous researchers have suggested that the intense ethical research frameworks involving young people in research studies can ultimately create obstructions to a process designed to have benefit for that participant group,^37^, ^38^, ^39^ and such obstacles may well be further exaggerated in an intervention study that has to consider the many ethical implications of an online intervention. These challenges include the way that adults negotiate access to children and the particular nature of the research environment (in the current study, the provision of online counselling through the parental home) in which researchers often engage with young people.^50^ We believe that the non‐targeted recruitment due to the ethical constraints of the study (necessitating a two‐phase process of consent before participants were confirmed as being eligible to take part in the study), alongside the failure to actively engage parental input in the planning process, may have created a significant barrier to recruitment. Anecdotal evidence from the parents of potentially eligible young people in the current study certainly suggested that parental reluctance, suspicion and concerns about engaging with the Internet and virtual environments created a barrier for recruitment into the study. Previous studies have suggested that greater parental knowledge about online interventions predicts more positive attitudes towards making use of such interventions for young people^51^ and highlights the need to not only better engage and educate parents about the potential benefits of online counselling in their role as key gatekeepers to increase uptake rates, but also in the actual design of the initial intervention.

Additionally, clinicians and nurses are a key and often trusted communication mechanism that can have a significant influence of study participation rates, with Gulliver *et al*.'s systematic review^52^ clearly identifying the importance of established and trusted relationships with professionals such as general practitioners as being an important facilitator in young people's help‐seeking behaviour. Patel *et al*.^53^ highlighted the need to engage school‐based, community‐based and specialist health professionals in supporting the provision of services addressing the mental health needs of young people to reduce reluctance to engage and fear of stigma. Whilst our study was supported through the local school, the funding charity and the NHS genetic service, it was not actively promoted by wider health professionals within the field of oncology and yet the latter are often seen as the key trusted gatekeepers in the cancer care process.^54^

## Conclusions

This study has demonstrated the complexities of intervention development and evaluation research targeted at young people and the many challenges created through attempts to merge the fields of clinical practice and research evaluation in a way that produces an ethically rigorous approach in terms of both practice and research without creating a research ‘obstacle course’ that creates too many barriers to successful recruitment and evaluation of the intervention. The process of engaging young people in the C:EVOLVE project enabled an appropriately targeted intervention to be developed and clearly demonstrated positive developmental opportunities for the young people through the creation of an environment for collaboration and intergenerational partnerships. The research team is confident that the online counselling intervention is now tailored appropriately for the target group, but the subsequent difficulties in recruiting for the evaluation phase of this project suggest that it is important to recognizing the parents’ ultimate gate‐keeping role in promoting or hindering the uptake of such interventions as well as the need to engage clinicians in the process of recruitment as trusted endorsers of the intervention in question. The research team is now keen to employ these lessons learned and is engaging in further in‐house exploratory work in relation to the usability of the intervention whilst seeking further funding to continue with Phase II recruitment to evaluate its potential therapeutic effectiveness and acceptability of the active components of the intervention.

## Funding

This project was funded by Tenovus the Cancer Charity under its Innovation Grant Scheme 2012 [TIG2012‐09].
